# Detecting Superior Face Recognition Skills in a Large Sample of Young British Adults

**DOI:** 10.3389/fpsyg.2016.01378

**Published:** 2016-09-22

**Authors:** Anna K. Bobak, Philip Pampoulov, Sarah Bate

**Affiliations:** Department of Psychology, Bournemouth UniversityPoole, UK

**Keywords:** face recognition, face perception, social anxiety, trait anxiety, super-recognizers

## Abstract

The Cambridge Face Memory Test Long Form (CFMT+) and Cambridge Face Perception Test (CFPT) are typically used to assess the face processing ability of individuals who believe they have superior face recognition skills. Previous large-scale studies have presented norms for the CFPT but not the CFMT+. However, previous research has also highlighted the necessity for establishing country-specific norms for these tests, indicating that norming data is required for both tests using young British adults. The current study addressed this issue in 254 British participants. In addition to providing the first norm for performance on the CFMT+ in any large sample, we also report the first UK specific cut-off for superior face recognition on the CFPT. Further analyses identified a small advantage for females on both tests, and only small associations between objective face recognition skills and self-report measures. A secondary aim of the study was to examine the relationship between trait or social anxiety and face processing ability, and no associations were noted. The implications of these findings for the classification of super-recognizers are discussed.

## Introduction

There are large individual differences in the ability to recognize (Bowles et al., 2009; Russell et al., 2009) and perceive (Megreya and Burton, 2006; Megreya and Bindemann, 2013) faces, and particular difficulties are associated with the processing of unfamiliar facial stimuli (see Hancock et al., 2000 for a review). These differences range from individuals who are remarkably good at face recognition (so-called “super recognizers,” SRs: Russell et al., 2009; Bobak et al., 2016a,b,c,d) to those affected by developmental prosopagnosia (DP; Bate and Tree, 2016). This latter group of people experience severe difficulties even when recognizing the most familiar of faces, in the absence of neurological damage or illness, lower-level visual or intellectual impairments or concurrent socio-emotional disorder (e.g., autism spectrum disorder) (Jones and Tranel, 2001; Bate and Cook, 2012; Susilo and Duchaine, 2013; Bate et al., 2014; Bennetts et al., 2015). Super-recognizers, on the other hand, outperform control participants on tests of face memory, and in some instances, face perception, (Russell et al., 2009; Bobak et al., 2016a,b,c).

The ability to recognize unfamiliar faces has also been found to be related to a number of personality factors. For instance, Bate et al. (2010) reported that people with high levels of empathy are better at recognizing newly learned faces than those who achieve lower scores on an empathy questionnaire. This may be because the additional information about others’ emotional state aids the encoding of new faces. Nonetheless, it is also possible that higher levels of empathy prompt perceivers to allocate more attention to faces. Another study by Hills et al. (2016) reported that psychometric schizotypy, a cluster of traits related to difficulties in social situations (e.g., anxiety), is negatively related to face recognition accuracy. Three studies have also shown a direct link between general anxiety and face recognition. In the first report, Mueller et al. (1979) divided participants into low and high anxiety groups and reported that those low in anxiety performed better in a face recognition task. Furthermore, Megreya and Bindemann (2013) demonstrated that neuroticism and anxiety are negatively correlated with face matching ability, but only in female observers. Finally, Davis et al. (2011) investigated the relationship between social and trait anxiety and the Cambridge Face Memory Test (CFMT), and found that poorer performance on the CFMT was correlated with a significant increase in participants’ social but not trait anxiety. These findings suggest that successful face recognition is associated with high socio-emotional functioning. This may be because gradual learning and the development of expertise is facilitated by the typical attention to faces that is present in non-anxious individuals. On the other hand, it is possible that those who are naturally worse at face recognition develop higher levels of anxiety due to consequent problems with social interactions. The former hypothesis is supported by studies showing that gregariousness is related to individuals’ face recognition ability through exposure (Li et al., 2010; Arnell and Dube, 2015) and that, conversely, shy children are less sensitive to cues necessary for face recognition (Brunet et al., 2010). However, only a longitudinal study would shed light on the developmental trajectory of the relationship between socio-emotional functioning and face recognition ability.

In order to assess superior face recognition, an extended version of the CFMT that is typically used to diagnose prosopagnosia (e.g., Duchaine and Nakayama, 2006; Duchaine et al., 2007), the CFMT+ (Russell et al., 2009), has been developed (see *Materials and Methods* for the full description of the test). In addition the Cambridge Face Perception Test, (CFPT, Duchaine et al., 2007) is frequently used to scrutinize the face perception skills of potential “super-recognizers”, mirroring the approach taken in the prosopagnosia literature where the CFPT serves as a supplementary measure of face processing (e.g., Duchaine et al., 2007). Just as some, but not all, individuals with prosopagnosia present with difficulties in face perception (e.g., Bobak et al., 2016d), it is plausible to assume that some SRs may present with superior face perception as well as face recognition skills. Russell et al. (2009) also used the “Before they were famous” test, in which participants view faces of celebrities that were taken some time before they became well known in the public domain. This diagnostic approach mirrors the technique that is typically used to diagnose DP, where participants are required to report long-lasting face recognition impairments and instances of an inability to recognize faces of their relatives, colleagues and famous people; score within the impaired range on the CFMT (≤42/72; Duchaine and Nakayama, 2006) and sometimes on the CFPT (≤60 mistakes on the upright trials, Duchaine et al., 2007), as well as show an inability to recognize famous faces on culturally specific sets of celebrity faces (Bate et al., 2014).

However, it is questionable whether tests born out of the prosopagnosia literature are suitably sensitive to detect superior performance at the top end of the face recognition spectrum, even with the additional “difficult” trials that are used in the CFMT+. The statistical approach used by Russell et al. (2009) in their seminal study identifying four SRs is also questionable, given they employed group-based analyses to compare the performance of these four individuals to those of a small number of controls (*N* = 25). Although the authors were attempting to apply standardized neuropsychological tests of face recognition ability to identify those at the top rather than the bottom end of the spectrum, unfortunately they failed to apply the standard statistical criteria that are typically used in neuropsychological diagnosis. That is, the cut-off of two standard deviations from the control mean is typically calculated to detect impaired or, in this case, superior performance on a case-by-case rather than a group basis (e.g., Schinka et al., 2010). When the sample size of the control group is ample, this technique is deemed appropriate. However, when the control group is small, many researchers use modified *t*-tests for single-case comparisons (Crawford and Howell, 1998) to provide a more conservative estimate of significantly different performance (e.g., Bobak et al., 2016a,b,c). This individual rather than group approach to diagnosis not only ensures that each individual participant meets the criteria for the condition in question, but is also of key importance when certain conditions are suspected to have cognitive heterogeneity, and the precise presentation of each individual has key theoretical implications. Much published work indicates this is the case in prosopagnosia and super recognition (e.g., Schmalzl et al., 2008; Bobak et al., 2016a).

As stated above, application of the standard neuropsychological approach of calculating two standard deviations from the control mean as a cut-off requires a substantial amount of control data to calculate these norms. In addition, there are likely to be different cut-offs on face recognition tasks according to standard demographic variables such as age, gender and even ethnicity (Bowles et al., 2009). This issue particularly applies to the CFPT given it incurs much more varied performance in controls, resulting in large standard deviations. In fact, when we applied the published norms for upright CFPT performance (Russell et al., 2012) to the scores of SRs in two published studies (Bobak et al., 2016a,d), it was near-impossible for these individuals to achieve significantly superior scores than the control group. This may be because the published norms are not suited to the demographics of the SR participants in these studies, but may also represent a more general issue with this test rendering it unsuitable for the detection of super recognition. Nonetheless, the CFPT may be useful for deriving an inversion index, a measure previously used by Russell et al. (2009) in their pioneering work on super recognition. Indeed, the effect of inversion is one of the best documented in face recognition research and is widely thought of as the hallmark of configural (or holistic) processing (e.g., Tanaka and Farah, 1993; Maurer et al., 2002).

Although the standard form of the CFMT has been shown to have very good psychometric properties making it suitable for the diagnosis of both acquired and developmental prosopagnosia (Duchaine and Nakayama, 2006; Bowles et al., 2009), barely any attention has been directed toward the suitability of the CFMT+ as a diagnostic tool in super recognition. The original paper identified four cases of SRs (Russell et al., 2009), but did not provide the control mean for either of its studies making comparison and direct replication of the procedure impossible. In the Russell et al. (2012) study the control (*N* = 26) mean and standard deviation were provided for the control group (*M* = 75.2, *SD* = 11.6) and the SRs (*M* = 95.0, *SD* = 1.9), but the individual scores of SRs were not reported, contrary to similar publications in the literature concerning DP. These papers usually provide individual face recognition scores so the reader can easily establish the number of standard deviations below the mean that an individual’s score is placed at.

In SR research, diagnosis to date has been based on a loosely defined cut-off point that is approximately two standard deviations above a specific control group’s mean. Using different control groups poses a risk in itself due to individual differences in performance. For instance, the control sample in Study 1 of Russell et al.’s paper appears to contain at least one, and potentially three (it is impossible to determine the exact score from the scatterplot), individuals who meet criteria for prosopagnosia. The prevalence of prosopagnosia has been previously reported to be 2–2.9% in an Australian sample (Bowles et al., 2009), 2.47% in a German young adult sample (Kennerknecht et al., 2006), and 1.2–4% in UK children (Bennetts et al., 2016). In the group of 25 control subjects in Russell et al.’s study, a prevalence rate of 3% would correspond to 0.75 participants. Given that the group potentially contained three individuals with DP, it may be that the control sample was atypical. Although it is possible that these individuals were not impaired in their face recognition performance, it is also plausible that they were affected by other neurodevelopmental disorders that are known to affect face recognition skills, such as autism spectrum disorder (see Weigelt et al., 2012 for a review).

Furthermore, the initial Russell et al. (2009) publication also provided a test of famous faces where participants are asked to recognize famous people from childhood photographs. The authors reported a strong positive correlation between the “Before They Were Famous” (BTWF) test and the CFMT and CFMT+; *r* = 0.70, *p* < 0.001 and *r* = 0.71, *p* < 0.001 respectively. These correlations, however, suffer from a sampling error that makes their meaningful interpretation difficult, if not impossible. Namely, within 29 subjects, four SRs make up 13.8% of the sample. This proportion is not representative of the general population where the prevalence of SRs, defined by performance of two standard deviation above the control mean, would not exceed 2.5%. Ultimately the top end of the distribution in the original report on SRs is artificially inflated and potentially confounds the correlational analysis presented in the paper. The conclusion that the BTWF test correlated with the CFMT should therefore be seen as tentative, at least until appropriate control data is published.

Finally, it is of note that SRs are typically identified via their own self-referral to a laboratory for testing. This tends to occur following media coverage of the phenomenon, where people suspect they may also have extraordinary face recognition skills. The issue of self-report has been contentious in the prosopagnosia literature, where most reports indicate that we have little insight into our face recognition skills (De Haan, 1999; Palermo et al., 2016; but see Kennerknecht et al., 2006; Shah et al., 2015). Notably, in their recent study, Palermo et al. (2016) compared the performance of 300 participants on a variety of behavioral measures against their self-reported face recognition ability. The authors argued that while those aware of their profound deficits in face recognition perform poorly on behavioral (objective) tests measuring this ability, typical perceivers have only modest insight into their face recognition skills. These findings are in contrast to the recent study by Shah et al. (2015) who reported strong correlations between their new questionnaire of face recognition ability (PI20) and the CFMT (Duchaine and Nakayama, 2006). Nonetheless, the latter report is a likely result of a statistical omission on the authors’ part. Specifically, the correlations presented in the Shah et al. (2015, Study 4) study were performed using a sample of participants that included typical perceivers and DPs. The DP participants constituted 17.2% of the overall sample – a prevalence that is highly unlikely to occur in any typical population (e.g., Kennerknecht et al., 2006; Bowles et al., 2009). If those with DP do have a greater insight into their abilities than typical perceivers, the inclusion of this special population could have artificially inflated the strength of the reported correlations. It is likely that the same findings apply to the self-report of superior face recognition skills, although no work to date has explored this issue.

It is also of note, that the CFMT and the CFPT have been previously reported to produce different average performance for individuals from various ethnic origins within the Caucasian population. Specifically, Bowles et al. (2009) reported that when the original norms (Duchaine and Nakayama, 2006) were employed, 8.9% of Australian participants were “diagnosed” as prosopagnosic, implying that for 119 volunteers tested in the study, eight were misdiagnosed as DP. Additionally, the authors showed that females performed better, albeit non-significantly, than males on the tests of face recognition and face perception. These findings further highlight the need for country- and perhaps, gender- specific norms for these two commonly used neuropsychological assessments.

The current study adopted the prevalent diagnostic technique that is used to identify SRs, and applied it to a large sample of 254 UK participants. We used the CFMT+, CFPT and self-report measures to assess the face recognition skills of the participants. The BTWF test was not used given its clear confounds that would be difficult to control within such a large-scale study. This approach allowed us to establish more reliable norms that match the approximate ages and nationality of the individuals tested to date. Given that application of the standard neuropsychological cut-off (i.e., two standard deviations from the control mean) results in the detection of the top or bottom two per cent of the population when a dataset is normally distributed, this approach also allowed us to assess the suitability of the tests for detecting potential SRs. This large dataset also permitted examination of the utility of self-report measures in detecting super recognition and, as a secondary aim, the ostensible relationship between face processing and trait and social anxiety.

## Materials and Methods

### Participants

Two hundred and fifty four participants (146 females and 108 males) with a mean age of 21.4 years (*SD* = 3.5, range 18–35) were recruited for this study, amongst students and visitors at Bournemouth University. The subjects were enrolled in several ways: through the online sign-up system for Psychology undergraduates, through advertisement at Bournemouth University and social media sites (i.e., Twitter and Facebook), and via opportunity sampling by the researcher (e.g., during science festivals and public engagement events). All volunteers were white Caucasians and were born (and spent the majority of their lives) within the UK. No participant reported any known history of brain injury or neurodevelopmental disorder that is likely to affect their face recognition skills (e.g., ASD, Weigelt et al., 2012). Most importantly, participants were not selected on the basis of their face recognition ability, but were simply recruited on a voluntary basis from the local population in Bournemouth. Overall, the education level in this sample was high due to the main site of recruitment (a higher education facility), reflected by the average number of years spent in full time education *M* = 15.1, *SD* = 2.2, range 11–26).

### Materials

#### Screening Questionnaire

An initial questionnaire enquired about general demographic information (e.g., age, gender, years of education) and any history of developmental disorders (e.g., Autism Spectrum Disorder or Williams syndrome), brain injury and periods of visual deprivation. Participants were also requested to provide self-ratings of their general face recognition ability, including estimates of instances where they have failed to recognize familiar faces, occasions where they have recognized faces only seen briefly or a long time ago, and their ability to follow characters on TV shows.

#### Cambridge Face Memory Test Long Form (CFMT+, Russell et al., 2009)

**FIGURE 1:**
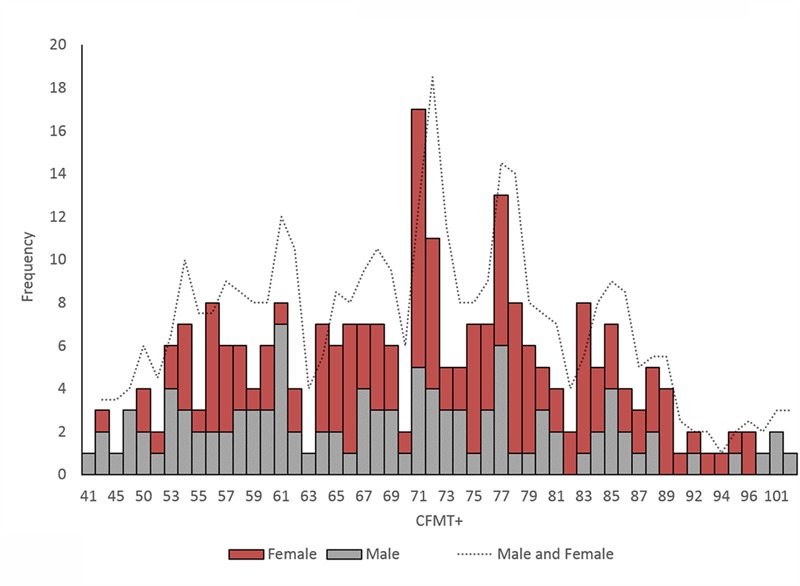
**Distribution of CFMT+ scores**.

This is an adapted version of the CFMT (Duchaine and Nakayama, 2006) with an added section of 30 “hard” trials containing heavily degraded images (see **Figure 1**). The original CFMT familiarizes people with six male faces. In the first section, subjects are presented with each face from three viewpoints and recognition of the same images is tested in a three-alternative forced-choice task. The second section is more challenging and the test images are novel and differ in pose and lighting conditions from the photographs used in the first part of the test. The third section also uses novel images, but with added visual noise which removes fine-grained information. While the CFMT (Duchaine and Nakayama, 2006) is commonly used to identify prosopagnosia and assess face recognition in the typical population (e.g., Bowles et al., 2009), it has a ceiling effect that makes it difficult to distinguish individuals with extraordinary face recognition memory. To address this issue, the fourth, additional section in the CFMT+ includes 30 trials of novel images varying in pose, emotional expression and the amount of information available (faces are fully cropped from the external features or presented with visible hairstyle and exposed neck). All photographs are heavily degraded using visual noise and the distractor identities recur more frequently than in the first three phases to minimize the difference in familiarity between the studied and distractor faces. These changes are thought to create a level of difficulty that is challenging enough to discriminate between people with typical but high face recognition memory and SRs.

#### Cambridge Face Perception Test

The Cambridge Face Perception Test (CFPT, Duchaine et al., 2007) requires participants to order six male faces in likeness to one target face. Participants complete eight upright and eight inverted trials with a time allowance of 1 min per set. In each trial participants are presented with a^3/4^; view image of the target face. The six test faces have been morphed to contain 88, 76, 64, 52, 40, and 28% of the target face. Responses for each trial are calculated based on the sum of deviations from the correct position of each face. For instance, if a face is sorted one position away from where it should be, this constitutes one error. If a face is four positions away, that is four errors. Errors for each trial are then summed to total upright and inverted deviations separately. Chance performance on the upright condition is represented by 93.3 errors.

#### State Trait Anxiety Inventory (STAI, Spielberger et al., 1983)

The STAI is a frequently used measure of state and trait anxiety in typical populations. It has internal consistency ranging from.86 to.95 and test-retest validity from 0.65 to 0.76 over a 2 months retention period (Spielberger et al., 1983). For the purpose of this study the trait anxiety scale (STAI-T) was used which included statements such as “I worry too much over something that doesn’t really matter” or reverse scoring questions such as “I am content; I am a steady person.” All items are scored on a four point scale with a maximum score of 80; a higher total score indicates greater trait anxiety.

#### Social Interaction Anxiety Scale (SIAS, Mattick and Clarke, 1998)

The SIAS comprises of 20 items which are rated by participants from 0 (not at all characteristic or true of me) to 4 (very characteristic or true of me). Items are statements describing one’s affective, cognitive and behavioral reactions to various situations (e.g., making friends, talking to people in positions of authority, or interacting at a party). There are three reverse scored items and the SIAS scores are summed up by adding the response values. The maximum score is 80 and higher scores indicate higher social anxiety in an individual. The scale has been validated for use in studies of social anxiety and social phobia and has good internal reliability (Cronbach’s alpha of up to 0.90, Heimberg et al., 1992; Mattick and Clarke, 1998).

### Procedure

All participants signed an informed consent form prior to the study. They were then invited to sit in testing cubicles and given the screening questionnaire including the STAI-T and SIAS scales. Subsequently, the CFMT+ and CFPT were administered. The researcher stayed in the room during instructions and practice trials to ensure that participants were comfortable with the tasks and able to ask any questions. Following the testing session, all participants were handed a debrief sheet, or debriefed verbally. Approval for this study was granted by the Bournemouth University Ethics Committee.

## Results

### CFMT+

The Kolmogorov–Smirnov test was used to test for normality on the CFMT+. The mean correct responses for the male group, *D*(108) = 0.08, *p* = 0.092, and the mean correct responses for the female group, *D*(146) = 0.07, *p* = 0.200, were both non-significant, indicating that the data was normally distributed in both gender groups. Analyzed together, the distribution of all CFMT+ scores was marginally abnormal, *D*(254) = 0.06, *p* = 0.049. This small departure from normality likely occurred because the significantly higher mean for female participants (see below) has created a trend toward a bimodal distribution when data is collapsed across genders. **Figure 1** illustrates the distribution of CFMT+ scores in males, females, and collapsed for all participants.

In order to explore potential gender differences in performance, scores on the CFMT+ were further subdivided to represent the number of correctly recognized faces in the four sections. It is possible that any differences that may arise between genders do so at the level where stable representations need to be formed (i.e., after the first familiarization phase that uses image, rather than face, recognition). This gave scores for the “same images” (section 1) out of 18 (chance performance = 6), for the “novel images” (section 2) out of 30 (chance performance = 10), for the “novel images with “noise” (section 3) out of 24 (chance performance = 8), and for the “difficult images” (section 4) out of 30 (chance performance = 10). For the purpose of analysis, the above sections are labeled as CFMT1 to CFM4, respectively. The whole test comprising of 102 trials is referred to as “CFMT+” and the short version of the task including the first three sections (72 trials) is referred to as “CFMT_72_.”

First a mixed analysis of variance (ANOVA) was conducted on the proportion of correct responses for the four sections of the CFMT+, with test block number as the within–participant factor (CFMT1/CFMT2/CFMT3/CFMT4) and gender as the between-participant factor (females/males). The analysis revealed a main effect of the CFMT test block, *F*(3,756) = 1010.63, *p* < 0.001, $\eta_{p}^{2}$ = 0.800; the main effect of gender, *F*(1,253) = 5.41, *p* = 0.021, $\eta_{p}^{2}$ = 0.021, with females performing better than their male counterparts, and a significant interaction between these two factors *F*(3,756) = 8.67, *p* < 0.001, $\eta_{p}^{2}$ = 0.033. Follow-up analyses of the within-subjects main effect (Bonferroni corrected) revealed a gradual decline in performance from CFMT1 to CFMT4 for all participants, all *p*s < 0.001.

Between-group *post hoc* analyses revealed that while there were no gender differences in performance on CFMT1 (*p* = 0.136) and CFMT4 (*p* = 0.569), females outperformed males on CFMT2 (*p* = 0.001) and CFMT3 (*p* = 0.012). Proposed cut-off points for SRs in young British adults are presented in **Table 2**. These were calculated by adding two standard deviations to the mean performance of the groups in the CFMT+ and subtracting two standard deviations in the CFPT.

### Cambridge Face Perception Test

A Kolmogorov–Smirnov test assessed normality on the main dependent variable: CFPT error rates. The mean error rates for the upright trials in the male group, *D*(108) = 0.13, *p* < 0.001, and the mean erroneous responses for the female group, *D*(143) = 0.13, *p* < 0.001, were both significant, indicating that the data was not normally distributed in both gender groups for the upright trials. The mean error rates for the inverted trials in the male group, *D*(108) = 0.07, *p* < 0.200, and the mean error number for the female group, *D*(143) = 0.07, *p* = 0.085, were both non-significant, indicating that the data was normally distributed in both gender groups for the inverted trials. Analysis of collapsed male and female data for the CFPT revealed that in the upright and inverted trials, data was not normally distributed, *D*(251) = 0.12, *p* < 0.001 and *D*(251) = 0.06, *p* = 0.015, respectively. Similarly to the CFMT+, this has likely occurred due to the bimodality of the distribution once the data are collapsed across genders.

A mixed ANOVA was conducted on the error scores, with trial type as the within–participant factor (upright/inverted) and gender as the between-participant factor (females/males). The analysis revealed a main effect of the trial type, in that participants were overall better at sorting faces in upright than inverted trials, *F*(1,249) = 1080.50, *p* < 0.001, $\eta_{p}^{2}$ = 0.813; and a significant difference between groups where females were better than males at sorting faces, regardless of orientation, *F*(1,249) = 6.82, *p* = .010, $\eta_{p}^{2}$ = 0.027. The interaction between these two factors was non-significant, *F*(1,249) = 3.08, *p* = 0.080, $\eta_{p}^{2}$ = 0.012 (see **Table 1** for mean and SD performance of the two groups).

**Table 1:** Gender effects on CFMT+ and CFPT scores for all participants.

	Gender of participants								
	Males			Females			Total		
	*N*	*M*	*SD*	*N*	*M*	*SD*	*N*	*M*	*SD*
CFMT1 (out of 18)	108	17.61	1.01	146	17.76	0.56	254	17.70	0.79
CFMT2 (out of 30)	108	21.76	5.69	146	23.98	4.41	254	23.04	5.11
CFMT3 (out of 24)	108	15.56	4.83	146	17.01	4.35	254	16.39	4.61
CFMT4 (out of 30)	108	13.78	4.60	146	13.47	3.90	254	13.60	4.21
CFMT+ (out of 102)	108	68.71	13.36	146	72.19	11.31	254	70.72	12.32
CFPT Upright	100	40.72	15.65	143	35.17	13.29	251	37.56	14.59
CFPT Inverted	108	69.39	13.50	143	67.07	13.16	251	68.07	13.33
CFPT Inversion effect	108	0.93	0.74	143	1.11	0.72	251	1.03	0.74

*CFMT, Cambridge Face Memory Test, CFPT, Cambridge Face Perception Test.*

**Table 2:** Proposed cut-off points for SR in young British adults.

Neuropsychological Test	Males		Females		Males and Females	
	C^∗^	N^∗∗^	C^∗^	N^∗∗^	C^∗^	N^∗∗^
CFMT+ (SR cut off)	95.43	4	94.81	2	95.36	6
CFPT Upright (SR cut-off)	9.42	0	8.59	0	8.38	0

*^∗^C, cut-off point on the neuropsychological assessment. ^∗∗^N, number of people in the sample that met these criteria.*

A final analysis was conducted on the CFPT inversion index for males and females. Each participant’s score for inverted trials was subtracted from their score for upright trials, then divided by their score for upright trials to create an inversion index ([upright-inverted]/[upright]; Russell et al., 2009). An independent samples *t*-test revealed a significant difference between the two groups, *t*(249) = 2.02, *p* = 0.44, *d* = 0.26, with females scoring a larger inversion effect than males (see **Table 1**).

### Do Young Adults Have Insight into their Face Recognition Ability?

Participants were also requested to provide self-ratings of their general face recognition ability, including estimates of instances where they have failed to recognize familiar faces, occasions where they have recognizing faces only seen briefly or a long time ago, and their ability to follow characters on TV shows. The responses to these questions were correlated with the CFMT+ scores of all participants (**Table 3**). These analyses revealed weak to moderate correlations between all variables. Of particular interest is the weak relationship between the CFMT+ scores and self-perceived face recognition ability, no relationship between the CFMT+ and the recognition of faces only seen briefly, and the highly significant relationship between the recognition of faces only seen briefly and self- reported face recognition ability. Additionally, we carried out an independent samples *t*-test to examine any gender differences that may arise in self-reported face recognition ability. However, there were no group differences in participants’ own estimation of their face recognition skills, *t*(252) = -0.211, *p* = 0.833, *d* = 0.03.

**Table 3:** The correlations between single items questions assessing self-perceived face recognition ability and the accuracy score for CFMT+ (*N* = 251).

Variables	1	2	3	4	5	6
(1) CFMT+	-					
(2) CFPT upright	-0.552^∗∗^	-				
(3) Self-reported face recognition ability	0.302^∗∗^	-0.257^∗∗^	-			
(4) Failure to recognize faces	-0.190^∗∗^	0.239^∗∗^	-0.379^∗∗^	-		
(5) Following characters in a movie	0.161^∗^	-0.135^∗^	0.420^∗∗^	-0.281^∗∗^	-	
(6) Recognition of faces seen briefly.	0.041	-0.055	0.287^∗∗^	-0.070	0.032	-

*^∗^Correlation significant at the 0.05 level. ^∗∗^Correlation significant at the 0.001 level.*

### Relationship with Anxiety

All participants filled in the SIAS and STAI-T inventories. The scores from these two questionnaires were correlated with objective and self-perceived face recognition ability. No relationship was observed between social and trait anxieties and the measures for unfamiliar face recognition in young British adults. Correlation coefficients of participants are reported in **Table 4**. It is also of note that while Davis et al. (2011) found a relationship between SIAS scores and the CFMT, the authors reported that these findings could not have been explained by individual differences in general cognitive ability. Whilst this study did not measure IQ directly, years of full time education of all participants were recorded in the initial screening questionnaire. In contrast to the results reported by Davis et al. (2011), years of education were negatively related to participants’ trait anxiety *r* = -0.214, *p* = 0.001, *N* = 254 and there was a trend in that better educated participants displayed less social anxiety than their less educated counterparts, *r* = -0.117, *p* = 0.080, *N* = 254.

**Table 4:** The correlations between single items questions assessing objective and subjective face recognition ability and trait and social anxiety (*N* = 251).

Variables	1	2	3	4	5
(1) CFMT+	-				
(2) CFPT Upright	-0.573^∗∗^	-			
(3) Self-reported face recognition ability	0.309^∗∗^	-0.284^∗∗^	-		
(4) Trait Anxiety	0.020	-0.002	-0.069	-	
(5) Social Anxiety (SIAS)	0.012	0.029	-0.076	0.688^∗∗^	-

*^∗^Correlation significant at the 0.05 level. ^∗∗^Correlation significant at the 0.001 level.*

Finally, the strong correlation between the STAI-T and SIAS scores suggest that the two scales tap into somewhat overlapping constructs and those participants who are generally anxious are also more likely to display high levels of more specific, social anxiety. Nonetheless, the rather far from perfect strength of correlation between these two measures suggests that social and trait anxiety are, at least in part, dissociable traits and should be investigated separately.

## General Discussion

This investigation aimed to examine the suitability of the current diagnostic criteria for the detection of super recognition. Using the two main tests that are currently employed for screening (the CFMT+ and the CFPT), together with self-report measures, relevant cut-offs were established using a much larger sample than in previous work. Additionally, this study set out to examine the ostensible relationship between anxiety and face processing ability. The importance of each of these issues is discussed in turn below.

### CFMT+

The CFMT+ emerged as a normally distributed measure without a ceiling effect – appropriate for classification of super recognition in young British adults. The previously published mean for this test was 75.2/102 (*SD* = 11.6) in a sample of 26 participants (gender unknown, Russell et al., 2012). The normative data for the sample of participants tested in this study is substantially lower by 4.48 points (see **Table 1**). This discrepancy could have occurred for a number of reasons. Firstly, the CFMT+ is comprised largely of a set of face images collected around the Boston area of the United States. Bowles et al. (2009) reported that the normative data for the short version of the CFMT in an Australian sample is much below the mean performance reported by Duchaine and Nakayama (2006) in their original paper. However, the performance of Israeli participants was on par with that reported in the original study (Duchaine and Nakayama, 2006). It is possible that the CFMT and ultimately CFMT+ are better suited to match a Southern European Caucasian sub-type, rather than to be used with participants of British or Northern European ancestry (Bowles et al., 2009). These results, in line with Bowles et al. (2009), further highlight the need for country-specific CFMT+ normative data.

One other explanation for this difference is the age of participants used in both studies. In Russell et al.’s (2012) study, the mean age of participants was 42.2 years, whereas the current study recruited young university students (mean age = 21.4). There is some evidence to suggest that face recognition ability matures late (Susilo et al., 2013) and it is possible that this difference in the CFMT+ performance reflects the age difference between the two samples. Nonetheless, this highlights the importance of using appropriately matched control samples in face recognition research.

Six individuals (two female) in the sample of young adults met the proposed criteria for SR classification on the CFMT+, amounting to 2.36% of the group. This is unsurprising, given that in normally distributed data, approximately 2.28% of cases fall outside the 2 SD cut-off. This limits the case that can be made that these individuals genuinely are SRs, as it simply identifies the top two per cent of the population, drawing into question our definition of super recognition. Pertinently, while at present the two SD cut-off is a working definition for super recognition, researchers should adopt multiple tests of face recognition to ensure that the same individuals consistently achieve high performance. It is also important to note, that while the literature commonly uses the two SD cut-offs for identifying both prosopagnosia (e.g., Bate and Cook, 2012) and super recognition (e.g., Russell et al., 2009; Bobak et al., 2016d), the cut-offs are rather arbitrary placeholders and should be merely used as guidelines until functionally meaningful thresholds can be determined.

In sum, the CFMT+, an extension of the widely used CFMT (Duchaine and Nakayama, 2006), emerged as a good assessment tool for super recognition, with a normal distribution of scores that are sufficient for the single-case approach commonly employed in neuropsychological research. Nonetheless, it is also apparent that well-matched control samples and extensive normative datasets are needed to draw meaningful conclusions from studies using this method of face recognition ability assessment.

### Cambridge Face Perception Test

The CFPT data in this study was not normally distributed and there was large variability in performance in both upright and inverted trials. One previous study using the CFPT in typical and SR participants (Russell et al., 2012) reported a similar mean score for the control sample (35.4) and the mean performance for SRs was 24.7 – a considerably different score from the cut-off point established in this study. Although their performance was better, the SRs in the study by Russell and colleagues still did not surpass the 2 SD below the control mean cut-off. This may be due to the specific structure, time constraints, and scoring of the test where a score involving only nine errors is simply impossible.

The precise process that is tapped by the CFPT is also unknown as the test does not resemble typical tasks that the human visual system encounters in real life. A difficult matching or sorting task may be more suitable to assess face perception in studies with typical perceivers and SRs.

Given (a) the narrow margin of error, (b) that the CFPT has been developed for studies with DP participants (Duchaine et al., 2007), and (c) that only group statistics have been reported in two previously published SR studies (Russell et al., 2009, 2012), we do not recommend this test for the screening of SR participants in future studies. Indeed, no participant scored more than 2 SDs above the mean in the current study. Instead, this test can serve as a non-binding guidance of participants’ face perception ability until more sensitive tests are developed.

### Self-Report

The initial decision to assess an individual for super recognition typically follows a self-report of extraordinary face recognition ability or referral by a member of family or friend (e.g., Russell et al., 2009, 2012). Research has long been interested in the reliability of self-reported face recognition aptitude (Kennerknecht et al., 2006; Bowles et al., 2009; Shah et al., 2015; Palermo et al., 2016) and to date all but one study (Shah et al., 2015) has showed that people have limited insight into their face recognition ability. The results from this study further corroborate these reports. Pertinently, self-reported face recognition ability was weakly, albeit significantly, correlated with the CFMT+ and CFPT. The accompanying three questions used to assess participants’ perceptions of their face recognition ability in various real-life situations also yielded weak or no correlations with the two objective measures of face processing ability used in this study. It is also possible that the relatively young age of the participants prevented them from having insight into their own abilities. Indeed, there is some evidence that face recognition ability matures late (Susilo et al., 2013) and it is possible that an older participant group would have better insight into how well they recognize faces. Researchers interested in this phenomenon may wish to investigate the influence of age on people’s judgments of their own face recognition ability.

In sum, these results show that people have limited insight into their face processing skills and that self-report should be treated with caution. However, it is also probable that the CFMT+ and the CFPT do not resemble real-life person recognition and the self-report measures would correlate better with applied tasks where there are more cues to recognition than the internal features. This is of particular importance to national security settings and the recognition of people from CCTV where, unless deliberately covered, the external features are typically available in an image. Future studies should endeavor to correlate self-report measures of face recognition with applied face processing tasks to examine this hypothesis.

### Relationship between Face Recognition Ability and Anxiety

In contrast to the previous findings (Davis et al., 2011), this study found no link between social or trait anxiety and face processing abilities in a large sample of young British adults. In addition to correlational analyses, individual analysis of the data from three participants who reported “much worse than average” face recognition ability revealed that none was significantly more anxious on an individual level (i.e., scored more than two standard deviations on SIAS or STAI-T) than the remainder of the sample.

These findings suggest that face recognition ability, both objectively measured and self-perceived, is not related to levels of social and general anxiety and it is plausible to assume that it does not affect every day social functioning. Given the low correlation between SIAS and CFMT (*r* = -0.177, *p* = 0.039) in the Australian sample, it is possible that the marginally significant result and the associated low effect size (explaining just above 3% of variance in the face recognition ability) is a false positive result. Alternatively, it is also probable that the Australian students are more aware of their face recognition impairments than young British adults, causing them to be more self-conscious of interactions involving other people. It is thus conceivable that the difference in self-awareness is underpinning the differences in the association between this study and results presented by Davis et al. (2011).

### Gender Effects

In two previous studies reporting gender effects in face recognition, it was found that females had a 2.5-point (Duchaine and Nakayama, 2006) and 2.7-point (Bowles et al., 2009) advantage in performance over their male counterparts on the CFMT task. In both studies these differences did not reach significance. In the sample reported here, females outperformed males by 3.48 points on their overall score on the CFMT+ (Russell et al., 2009). Pertinently, these differences emerged in the novel stages of the CFMT+ (CFMT2 and CFMT3) but did not extend to the more difficult CFMT4. These discrepancies could have emerged for a number of reasons. Firstly, in CFMT1 participants are asked to recognize six faces from identical images to those presented in the study phase for each of the identities. This task relies on image matching rather than face recognition ability, using pictorial representations created throughout the study phase. It is likely that this relatively easy section has a ceiling effect for both males and females, obscuring any differences in performance. In contrast, CFMT2 and CFMT3 test actual face memory by presenting novel views of the identities, requiring activation of the view-independent representations created throughout the familiarization period. It is possible that females are more proficient at creating these view-independent representations following a restricted number of exposure times. What is more, although the test faces in CFMT3 are partially blurred by the overlay of visual noise, the configural and fine-grained details of all faces are largely available. This is not the case in CFMT4, and it is possible that the heavy visual noise is obstructing the detection of face configuration *per se* and as such makes identity judgments near impossible for the typical perceiver. As such, the lack of gender difference in the last section of the test may be due to the apparent floor effect in this section. It is thus likely that the fourth part of the CFMT+ is more suited to assessing the initial level of face detection, rather than higher order relations necessary to extract identity. This process may not be influenced by participant gender. In contrast, it is possible that females process faces more holistically than males, which would have aided performance on CFMT2 and CFMT3. Pertinently, there was a significant difference, albeit with a small effect size, between participants on the CFPT inversion effect, adding support to this hypothesis. Future research may wish to investigate this possible mechanism underpinning the female advantage in face recognition ability.

Importantly, there was also a significant, albeit small, advantage for females over males in the CFPT. Similarly to the Bowles et al. (2009) report, this minor gain was combined with a somewhat smaller variability in the female group. Nonetheless, this produced merely a one point difference in the proposed cut-offs and as such, the strict gender match of control participants to a SR sample is not necessary.

The results from this study are the first to indicate that females are significantly better than males at both face recognition memory and face perception. This finding stands in contrast to previous work reporting an advantage for females in the recognition of female, but not male, faces (McKelvie et al., 1993; Lewin and Herlitz, 2002) and weakens the meaningfulness of the “own-group bias” (Bernstein et al., 2007) explanation for this effect. Indeed, young British females outperformed males at the CFMT+ and the CFPT tasks, which both only use male faces. While there are no equivalents of these two tests utilizing female faces as stimuli, it is plausible that the sex difference would have been even more pronounced for CFMT+ and CFPT if female faces were used, thus providing support for the “own-group” hypothesis. Future work should devise face recognition assessment batteries utilizing male and female faces to further elucidate this issue.

It is important to mention that while the sample of participants in this study was not selected according to face recognition ability, the participants were recruited opportunistically rather than randomly. It is thus not improbable that the groups differed in their face recognition ability rather than this finding representing a true gender difference. Nonetheless, a seminal study investigating face recognition ability in Australian and Israeli samples (Bowles et al., 2009) showed a similar, albeit non-significant, result, indicating a small advantage in face recognition ability for females. Future work should further investigate the issue of gender effects in face processing using standardized assessments and wide range of stimuli.

## Conclusion

Taken together, this study further highlights the importance of establishing the norms in the country of origin for the purpose of neuropsychological research and is the first to show significant gender differences in face recognition memory and face perception in a sample of young adults. The data reported here suggests that face recognition ability is largely normally distributed and that it is plausible to assume that DPs and SRs represent the opposite ends of one continuum. It is thus recommended that future studies in face processing use appropriate ethnic and gender-matched samples in order to draw meaningful conclusions from data. This study also provides converging evidence that we have limited insight into our face recognition ability. Further, it is possible that the CFMT+ and the CFPT are not suitable for assessing real-world face recognition skills at the top end of the spectrum, and more applied tasks may be needed to assess the true extent of people’s face recognition ability. Finally, no link was found between behaviourally measured and self-reported face recognition ability and trait and social anxiety. Future studies should examine the possible link between self-awareness of one’s cognitive abilities and these personality characteristics.

## Author Contributions

AB designed the study, collected and analyzed the data and wrote the manuscript jointly with SB. This study is a part of AB’s doctoral thesis. PP recruited participants, collected data and wrote sections on social and trait anxiety. SB is a joint senior author, was involved in the design of the study, participant recruitment, data analysis and write-up of the paper. SB is AB’s doctoral supervisor.

## Conflict of Interest Statement

The authors declare that the research was conducted in the absence of any commercial or financial relationships that could be construed as a potential conflict of interest.
